# Bioengineered In Situ-Forming Hydrogels as Smart Drug Delivery Systems for Postoperative Breast Cancer Immunotherapy: From Material Innovation to Clinical Translation

**DOI:** 10.3390/jfb16100381

**Published:** 2025-10-10

**Authors:** Yan Yan, Yiling Chen, Litao Huang, Menghan Cai, Xia Yin, Yi Zhun Zhu, Li Ye

**Affiliations:** School of Pharmacy and Laboratory of Drug Discovery from Natural Resources and Industrialization, Macau University of Science and Technology, Macau SAR, China; 3220006860@student.must.edu.mo (Y.Y.); ylchen@must.edu.mo (Y.C.); lihuang@must.edu.mo (L.H.); 3220006407@student.must.edu.mo (M.C.); yinxia32014@163.com (X.Y.); yzzhu@must.edu.mo (Y.Z.Z.)

**Keywords:** breast cancer, in situ-forming hydrogels, immunotherapy, local recurrence, postoperative care, translational research

## Abstract

Local recurrence after breast cancer surgery presents a critical challenge, demanding novel local immunotherapies capable of eliminating residual disease while avoiding systemic toxicity. In situ-forming hydrogels, functionalized with bioactive cargoes, represent a promising platform for precise spatiotemporal drug delivery directly into the post-resection tumor microenvironment. This review comprehensively examines the core design principles governing these advanced materials, highlighting their biocompatibility, stimuli-responsive behavior, tunable mechanics for conforming to surgical cavity, and capacity for multifunctional integration. A key mechanism discussed is how this controlled release profile orchestrates a temporal progression from innate immune activation to robust adaptive immunity. Despite significant promise, translational success faces substantial hurdles, including efficacy validation, scalable manufacturing, regulatory pathway definition, and the lack of predictive biomarkers. Future research priorities include optimizing drug/antigen release kinetics, establishing standardized characterization methods for complex biohybrid systems, and designing adaptive clinical trials incorporating detailed immunomonitoring. By integrating functional biomaterials with immuno-oncology, in situ-forming hydrogels offer a paradigm-shifting approach for postoperative cancer treatment. This review provides a strategic roadmap to accelerate their translation from bench to bedside.

## 1. Introduction

### 1.1. Clinical Imperative and Current Limitations

A significant challenge persists in the management of breast cancer: despite advances in early detection and systemic therapy, up to 30% of early-stage patients experience recurrence within a decade [1]. This persistent failure stems from residual tumor cells surviving in the immunosuppressive microenvironment created by surgical trauma—a paradox where curative resection inadvertently fosters metastatic progression [2,3]. Conventional adjuvant therapies present limitations: chemotherapy induces acute toxicity and long-term organ dysfunction [4], endocrine therapy is hindered by poor patient compliance [5], and radiation carries secondary malignancy risks [6]. Even targeted agents fail to eliminate minimal residual disease (MRD) in a sustained manner due to intratumoral heterogeneity and adaptive resistance [7]. The absence of a survival benefit for aggressive surgery over breast-conserving techniques underscores an urgent need for localized strategies that selectively target MRD while preserving quality of life [8].

### 1.2. In Situ-Forming Hydrogels as a Paradigm-Shifting Solution

Bioengineered in situ-forming hydrogels offer a transformative approach to addressing the above limitations by redefining the post-resection cavity as a site for localized therapeutic intervention. Unlike traditional systemic therapies, which are limited by off-target toxicities and suboptimal tissue penetration [9], these intelligent biomaterials directly transition from liquid precursors to structured matrices within the surgical bed [10,11], offering distinct advantages over alternative hydrogel-based drug delivery systems. A defining advantage of in situ-forming hydrogels lies in their ability to mold seamlessly to irregular post-resection cavities, eliminating the mechanical trauma associated with pre-shaped implants. In this respect, Mi et al. demonstrated that photopolymerizable poly(ethylene glycol) dimethacrylate/sericin methacryloyl hydrogels conformally filled mouse mammary resection cavities, forming a physical barrier that minimized drug leakage and achieved ~50% cure rates in tumor-bearing mice, with survivors remaining disease-free for over six months [12]. Similarly, Zhang et al. engineered oxidized sodium alginate-tumor cell membrane vesicle hydrogels that self-assembled in situ to modulate T cell exhaustion and MHC-I expression locally [13]. In contrast, static hydrogels or rigid scaffolds often fail to match complex geometries, leading to uneven drug distribution and suboptimal tissue integration [14]. This ensures maximal contact with residual disease while avoiding secondary injury from implantation.

Interestingly, the dynamic crosslinking kinetics of in situ-forming hydrogels enable the precise tuning of drug release profiles, which is critical for exploiting the narrow window of post-surgical immune plasticity. In this context, Ziaei et al. exploited thiolated polyvinyl alcohol/poly(ethylene glycol) diacrylate hydrogels that gelled within seconds, enabling sustained doxorubicin release and enhancing local chemoimmunotherapy efficacy [15]. Such temporal precision surpasses passive diffusion-driven release from pre-fabricated hydrogels, which often result in premature burst release or delayed payoff beyond the immune-sensitive period [16].

In situ-forming hydrogels transcend unimodal therapy by serving as multifunctional hubs for combinatorial immunomodulation. For instance, Dong et al. chemically crosslinked M13 phage-displaying hydrogels in situ to reverse immunosuppression and significantly enhance the anti-breast cancer response [17]. Wang et al. advanced this concept using peptide-based supermolecular silk hydrogels co-delivering anti-PD1 antibodies, IL-15, and STING agonists, achieving tumor regression and overcoming monotherapy resistance [18]. These platforms outperform modular approaches (e.g., layered hydrogels or separate nanoparticle formulations) by enabling spatiotemporally coordinated co-release of antigens, adjuvants, and immune modulators—a feature critical for breaking tumor heterogeneity and immunosuppressive barriers [18,19]

### 1.3. Mechanistic Advantages of Gel Systems

**Figure 1:**
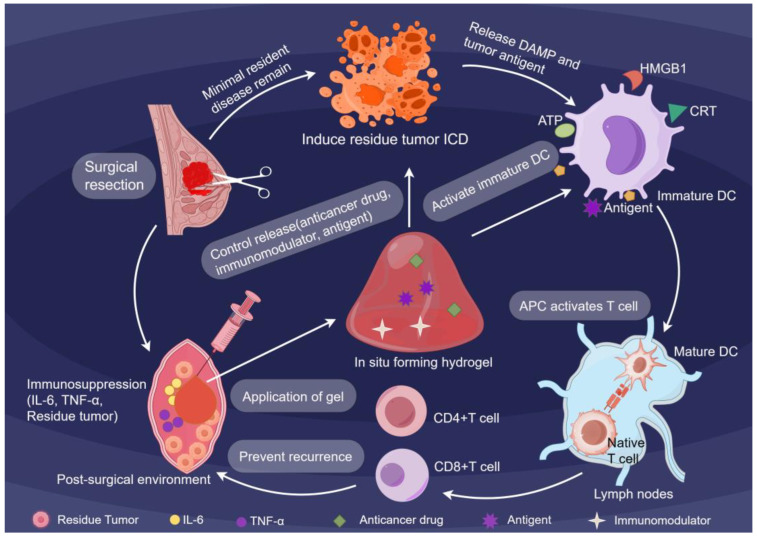
Schematic illustration of the mechanism of action of an in situ forming hydrogel within the post-resection cavity (generated using Figdraw 2.0).

The efficacy of this platform lies in its sophisticated material design. Modern hydrogels are strategically designed with adjustable properties, including crosslinking density, degradation kinetics, and mechanical compliance, to coordinate with the timing of tissue repair and immune activation [20,21].Building upon these physical foundations, the hydrogels execute a multistep immune-engineering cascade (Figure 1). By occupying the surgical cavity, they physically block pro-fibrotic signaling while releasing immunostimulatory cargoes, effectively converting the resection site into a center that drives systemic antitumor immunity [22]. Simultaneously, the hydrogels leverage their tunable degradation profiles to actively release ICD inducers, driving immunogenic cell death (ICD) in residual tumor cells. This triggers the release of damage-associated molecular patterns (DAMPs), including CRT, ATP, and HMGB1, transforming dying tumor cells into endogenous antigen reservoirs. Consequently, these synergistic signals collectively break immune tolerance and activate naive T cells to target MRD [23]. This strategic localization enables selective targeting of minimal residual disease while preserving healthy tissue, thereby maintaining patient quality of life.

### 1.4. Evolution and Clinical Progress of In Situ-Forming Hydrogels

Over the past two decades, in situ-forming hydrogel technologies have evolved from passive carriers to multifunctional platforms integrating stimuli-responsiveness, cell-immune modulation, and real-time monitoring capabilities [24,25]. Hu et al. developed an injectable chitosan/β-glycerophosphate (β-GP) thermosensitive hydrogel that underwent in situ gelation upon exposure to physiological temperatures, forming a depot for sustained therapeutic agent release. This system achieved long-term synergistic efficacy when combined with post-tumor-resection immunotherapy [22]. Jaiswal et al. engineered a silk-based hydrogel promoting angiogenesis and modulating macrophage polarization to enhance localized drug delivery and tissue regeneration following breast cancer surgery [26]. Interestingly, contemporary hydrogel designs incorporate pH-sensitive linkers for lysosomal escape and matrix metalloproteinase (MMP)-degradable domains for T cell co-localization, thereby achieving synergistic therapeutic effects [27,28].

Notably, while these advances underscore the translational potential of in situ-forming hydrogels, there are currently no active or planned clinical trials specifically evaluating their application in the postsurgical breast cancer setting (as confirmed by ClinicalTrials.gov search on 26 September 2025). This contrasts sharply with progress in other malignancies: for example, UGN-102—an in situ-forming hydrogel approved for intravesical chemotherapy in bladder cancer demonstrating safety and sustained drug release [29]. Similarly, A thermosensitive hydrogel loaded with paclitaxel—OncoGel^®^—has completed early Phase I/II clinical trials in various solid tumors, including superficial solid tumors and esophageal cancer [30]. Additionally, a cholesterol-based pullulan (CHP) nanogel vaccine has been evaluated in Phase I/II trials among patients with advanced solid tumors such as esophageal cancer, prostate cancer, and melanoma [31].

These non-breast cancer studies validate core functionalities (localized delivery, tumor bed coverage) but highlight the urgent unmet need for breast cancer-specific trial designs accounting for unique mammary stroma biology.

Ultimately, in situ-forming hydrogels represent a transformative convergence of bioengineering and immuno-oncology, redefining the post-resection cavity as a programmable immune niche. By integrating material innovation with immunological precision, they resolve the paradoxical dichotomy between curative intent and immunosuppression through localized, spatiotemporally controlled immunomodulation. Overall, this review outlines how smart hydrogels can transform cancer care by repurposing the surgical site to generate antitumor immunity. Key advances in their design and preclinical development are synthesized to provide a framework for clinical translation

## 2. Material Innovation: Rational Design of Bioengineered Hydrogels for Controlled Immunotherapeutic Delivery

The success of postoperative breast cancer immunotherapy fundamentally depends on the ability to engineer biomaterial platforms that act as intelligent intermediaries between therapeutic intent and biological reality. This section examines the rationale behind material innovation, specifically how bioengineered hydrogels are designed to address the distinct challenges posed by the tumor resection microenvironment. An analysis of essential design parameters is undertaken, encompassing the critical requirements of biocompatibility, stimuli-responsive behavior, adjustable mechanical characteristics, swelling capacity, and hydrophilicity. In addition, the dynamics of gelation tailored to specific anatomical needs are discussed, along with the integration of multifunctional immune modulators, and strategies for surface engineering that help overcome barriers to biocompatibility.

### 2.1. Core Design Principles

The rational design of bioengineered hydrogels for postoperative breast cancer immunotherapy relies on five key pillars: biocompatibility, stimulus-responsiveness, mechanical tunability, swelling capacity, and hydrophilicity. These principles govern the selection of materials and dictate the functional performance of in situ-forming systems in the dynamic tumor resection microenvironment.

#### 2.1.1. Biocompatibility as a Foundation with Breast Postoperative Implications

The selection of hydrogel backbones has a critical influence on immunomodulatory outcomes, with both natural and synthetic polymers offering complementary benefits. Natural polymers, such as chitosan, hyaluronic acid (HA), and alginate, are often prioritized for their intrinsic bioactivity and biocompatibility; however, their utility must be evaluated against breast-specific postoperative constraints. Chitosan’s inherent cationic nature activates the STING pathway via immune cell interactions [32], yet its formulation requires surface engineering to ensure it neither compromises local immune programming nor hinders epithelialization. Similarly, HA engages CD44 receptors on residual cancer cells to release DAMPs, thereby amplifying innate immunity [33,34]. For clinical suitability, these materials demonstrate hemocompatibility and a degradation profile that minimizes pro-inflammatory responses, thereby mitigating complications such as seroma formation. Alginate-based systems further enhance therapeutic efficacy by enabling the mild encapsulation of chemotherapeutics such as doxorubicin [35]; however, they require buffering modifications to neutralize acidic degradation byproducts that could exacerbate inflammation or impair protein stability.

Conversely, synthetic polymers such as poly(lactic-co-glycolic acid) (PLGA) and polyethylene glycol (PEG) offer tunable release kinetics and structural stability, although their degradation behavior requires rigorous optimization for breast applications. While PLGA’s degradation rate is determined by its lactide: glycolide ratio [36], its acidic metabolites necessitate buffering strategies to prevent tissue irritation and preserve wound microenvironment homeostasis [37].

Among synthetic polymers, PEG hydrogels serve as a foundation for modern biomaterial design due to their exceptional biocompatibility and versatility—attributes particularly critical for breast cancer postoperative care. PEG’s neutral charge, high hydrophilicity, and resistance to nonspecific protein adsorption inherently minimize adverse host responses, establishing it as an FDA-approved gold standard for clinical applications [38]. These properties directly address breast-specific surgical challenges, support safe interaction with the wound bed. Crucially, PEG’s tunable molecular weight and chain architecture enable precise control over crosslinking density and swelling ratios—parameters engineered to avoid excessive fluid retention or irritant acidic degradation byproducts, thereby reducing seroma formation and postoperative inflammation [39]. This balance between bioinertness and functional adaptability has recently been demonstrated in enzyme-responsive PEG nanogels, where MMP-cleavable crosslinkers enabled the targeted release of immunomodulators while maintaining the material’s favorable biocompatibility profile [40].

Although significant progress has been made in developing biocompatible natural and synthetic polymers, hydrogel degradation byproducts or residual monomers may still function as pro-inflammatory stimuli. Future advancements require the development of more precise monitoring technologies to coordinate the spatiotemporal dynamics between degradation rates and inflammatory regulation [41].

#### 2.1.2. Stimuli-Responsiveness for Spatiotemporal Control

**Figure 2:**
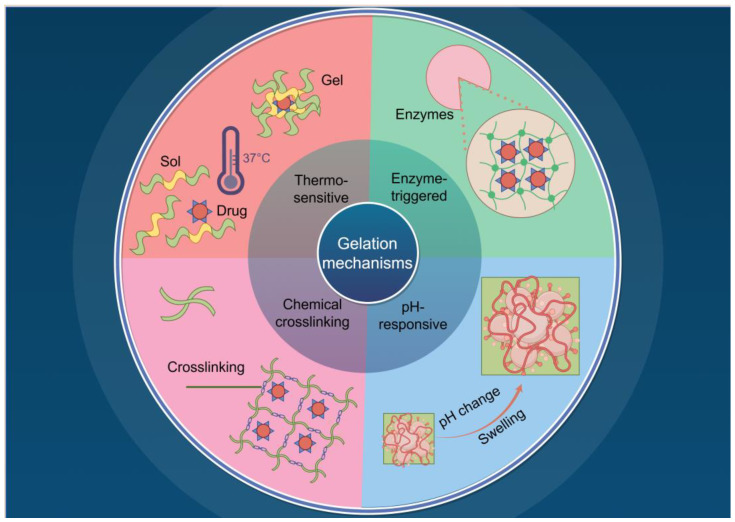
Four fundamental gelation mechanisms in in situ gelation: thermo-sensitive, enzyme-triggered, chemical crosslinking, and pH-responsive (generated by Figdraw 2.0).

Precise spatiotemporal control of immune activation demands hydrogels that dynamically respond to physiological cues. In Figure 2, four key gelation types are presented: thermosensitive, pH-responsive, chemically crosslinked, and enzyme-triggered systems, each defined by specific activation mechanisms and adapted for clinical purposes. For instance, thermosensitive hydrogels, such as poly (N-isopropylacrylamide) and Pluronic F-127, undergo a rapid sol–gel transition upon injection, facilitated by body heat [42]. pH-responsive systems leverage groups such as carboxyl and amino moieties that undergo structural modifications in acidic tumor microenvironments, thereby supporting site-specific drug delivery [43]. Advanced platforms integrate multiple stimuli, such as hybrid crosslinking networks combining thermal and ionic triggers, to maintain gelation reliability across variable postoperative temperatures [44]. Enzyme-responsive hydrogels, including MMP-cleavable matrices, harness tumor-specific enzymatic activity for on-demand drug release [45], while ROS-sensitive formulations amplify oxidative stress for localized therapy [46]. Collectively, these responsive mechanisms enable adaptive immunomodulation precisely tuned to the unique conditions of pathological microenvironments.

#### 2.1.3. Mechanical Tunability for Surgical Compatibility

Hydrogels for the breast lumpectomy cavity must strike a balance between mechanical resilience and tissue-like compliance to address the biomechanical complexities of the surgical site. Ouyang et al. addressed this challenge by engineering hydrogen-bonded poly(hydroxyethyl methacrylate) hydrogels incorporating maleic acid units, which exhibit elasticity and toughness comparable to that of native breast tissue [47]. Zheng et al. further advanced this paradigm by adapting the silk fibroin/mesoporous bioactive glass/sodium alginate composite to accommodate local Ca^2+^ or pH fluctuations, thereby ensuring sustained performance amid the dynamic biochemical shifts of the evolving wound microenvironment [48].

Shear-thinning behavior enables non-invasive injection, while rapid recovery moderately dissipates periodic shear strains from respiratory movements, allowing adaptation to the dynamic breast cavity environment [49]. Co-crosslinked HA-silk fibroin hydrogels exemplify this balance, combining rapid cavity filling with strong tissue adhesion to prevent intraoperative sticking [50]. These innovations systematically address the biomechanical mismatch between synthetic materials and soft tissue interfaces, ensuring clinical translatability. By prioritizing deformation recovery, surface adhesion under wet/lipid conditions, these mechanically tunable hydrogels transform theoretical advantages into practical solutions for breast cancer surgery.

#### 2.1.4. Swelling Capacity: Orchestrating Drug Release

To control the therapeutic cargo loading and release within the breast lumpectomy cavity, the swelling capacity of the hydrogel must be precisely engineered. Increasing crosslink density markedly restricts swelling by constraining polymer chain mobility and reducing network porosity, thereby limiting water penetration [51]. This property is critical in the early postoperative phase, where uncontrolled swelling risks occluding closed-suction drains or displacing the hydrogel matrix. Conversely, a low crosslink density increases mesh size, enabling a burst release that is ideal for immediate immune activation while balancing fluid handling to manage exudate and reduce seroma formation [52,53]. For instance, Khan et al. demonstrated that high-swelling hydrogels at pH 1.2 achieved maximal drug release via enhanced porosity; however, such behavior must be tempered in vivo to prevent premature payload depletion [54]. Denser crosslinking prioritizes structural stability over swelling, favoring sustained release profiles essential for prolonged antigen presentation or checkpoint inhibitor delivery [55]. The past few years have witnessed the advent of drug delivery systems that leverage stimulus-responsive swelling. In this respect, Jensen et al. engineered GelMA/SPA composites that cyclically expand/contract with ionic strength shifts, enabling on-demand release synchronized to postoperative inflammation waves [52], while Murugesan et al. fine-tuned hyaluronic acid hydrogels to balance shape stability and diffusivity, preventing dilution in the wet cavity while sustaining staged release across healing timelines [56].

#### 2.1.5. Hydrophilicity: Governing Cell–Material Interactions at the Interface

The hydrophilic character of hydrogels, defined by their three-dimensional networks containing polar functional groups (e.g., –OH, –COOH), governs critical cell–material interactions at the breast lumpectomy cavity interface [57]. By modulating crosslink density, surface chemistries, or amphiphilic block ratios, researchers can precisely tune the hydrophilic–hydrophobic equilibrium to balance stable adhesion on lipid-contaminated adipose–fibrous tissue with cellular compatibility—a necessity for maintaining intimate contact with residual mammary epithelium and immune cells. Butenko et al. systematically varied crosslink density in gelatin-based hydrogels to probe its impact on cell behavior. Their findings revealed that highly crosslinked, relatively hydrophobic matrices promoted fibrogenic fibroblast activation via the RANKL signaling pathway, subsequently driving macrophage fusion and fibrotic responses [58]. Conversely, surfaces enhanced for hydrophilicity preferentially recruited anti-inflammatory M2-polarized macrophages, thereby suppressing excessive scarring while promoting ordered stromal integration [59].

### 2.2. Gelation Mechanisms Tailored to Surgical Cavity Anatomy

The clinical efficacy of in situ-forming hydrogels relies on their ability to precisely conform to the irregular topography of mastectomy cavities while maintaining structural integrity. While the theoretical framework presented in Figure 2 outlines design principles for the fundamental crosslinking mechanism, effective clinical application necessitates a strong adaptation to the realities encountered during surgery. This is critical within the hypothermic tumor bed, where standard gelation kinetics may prove inadequate.

#### 2.2.1. Rapid Gelation Kinetics in Cooled Resection Cavities

The hypothermic microenvironment of post-resection cavities imposes stringent requirements on gelation kinetics, demanding rapid phase transition without compromising structural fidelity. Tyramine-conjugated HA hydrogels overcome this challenge by harnessing reactive oxygen species for instantaneous crosslinking, thereby maintaining functionality even at temperatures below physiological levels [60]. Complementarily, dual-triggered systems integrate thermal responsiveness with ionic crosslinking, ensuring reliable gel formation across temperature gradients encountered during surgical procedures [44]. These innovations address the critical bottleneck of material performance in non-physiological environments. While these engineered systems demonstrate robust performance under controlled conditions, interpatient variability in cavity temperature profiles—dictated by ambient operating theater conditions, and individual thermoregulation—challenges the universal applicability of fixed gelation parameters. Future efforts must therefore prioritize tunable reaction kinetics, biocompatible catalyst systems, and real-time monitoring of gelation progression in situ to achieve an optimal balance between speed and safety without compromising reliability [61].

#### 2.2.2. Multifunctional Cavity Coverage

The adipose-fibrous tissue constitution and significant deformation of the postoperative breast cavity make “tight, durable, and controllable” interface coverage particularly critical. Asymmetric adhesion combined with dynamic surface adaptation enables strong tissue-side attachment while preventing adhesions on the lumen side, thereby achieving uniform encapsulation of complex geometries [62]. Furthermore, chitosan-based hydrogels, leveraging their cationic surfaces for mucosal-like adhesion, establish stable contact at moist, lipid-rich surgical margins. Simultaneously, they provide a favorable microenvironment for cell adhesion and proliferation, promoting wound healing through enhanced re-epithelialization and granulation tissue maturation [63,64,65].

Thermo/pH-responsive chitosan–glycerophosphate systems demonstrate exceptional adaptability, given that they can be injected and gel in situ to conformally cover irregular margins, enabling on-demand permeability for molecular transport [66]. This dynamic behavior ensures sustained interaction with remodeling tissues, as demonstrated by studies showing accelerated wound healing and support for transplanted mesenchymal stem cells in vivo [65]. Recent developments in multiresponsive chitosan nanohydrogels extend these capabilities, with pH-sensitive cargo release under tumor-like acidic conditions and positively charged surfaces that favor stable tissue interfacing and cellular interactions [67].

These integrated strategies collectively transform the resection cavity into a structurally optimized, conformal therapeutic interface that supports sustained immunotherapeutic intervention. By leveraging dynamic interfacial adaptation, thiourea-catechol crosslinked hydrogels achieve conformal coverage through synchronized material deformation and tissue remodeling, acting as biodegradable dressings that progressively align with healing tissues [68]. Zhou et al. developed a nanocomposite hydrogel exhibiting asymmetric adhesion: one surface adheres tightly to tissue, while the other resists adhesion, enabling uniform coverage over complex anatomical geometries [69]. Further refinement is achieved through the use of alginate derivatives with tunable phenolic hydroxyl densities, which facilitates the modulation of adhesion strength to match diverse surgical contexts [70]. Together, these multifunctional designs harmonize material properties with biological responses, thereby transforming the breast cancer resection cavity into a therapeutic interface adaptable to tissue remodeling.

### 2.3. Multifunctional Integration of Antigens, Adjuvants, and Immune Modulators

Hydrogels function as advanced immunological platforms by precisely coordinating the spatiotemporal delivery of antigens, adjuvants, and immune modulators with the dynamic processes of immune cell activation and maturation.

#### 2.3.1. Design–Function Correspondence

Release kinetics and immunogenicity are determined by three loading strategies: physical entrapment, affinity-based binding, and covalent conjugation. Physical entrapment within layered poly(arylsulfone) reservoirs enables passive diffusion-based release, though rapid kinetics necessitate the use of retention enhancers [71]. Affinity-mediated loading, exemplified by chitosan-alginate hydrogels, stabilizes therapeutic cargo via electrostatic interactions, thereby prolonging immune activation [72]. Covalent conjugation via redox-cleavable bonds improves dendritic cell uptake and epitope presentation, albeit with potential conformational constraints [73]. Advanced synergistic architectures integrate multiple modalities, such as physical entrapment combined with affinity domains. This integration achieves a temporal balance that allows for an initial burst release, facilitating rapid recruitment of antigen-presenting cells (APCs), while also ensuring sustained exposure that is crucial for clonal expansion [74]. This multimodal integration enables precise spatiotemporal control over immunomodulatory release, thereby fine-tuning the kinetics of antigen presentation and immune cell activation.

Despite these advances, significant challenges remain in translating controlled release profiles into therapeutic outcomes. Especially for in situ hydrogel platforms, preventing premature diffusion or degradation while maintaining antigens persistently remains a challenge. Concurrently, hydrophilic hydrogels inherently struggle to retain hydrophobic antigens, resulting in burst release and suboptimal T cell priming [75]. This highlights the need for sophisticated design strategies that strike a balance between retention and controlled release.

#### 2.3.2. Co-Localization for Immune Priming

Effective immune priming necessitates the precise spatial alignment of antigens, danger signals, and costimulatory cues. Tumor microenvironment targeting prioritizes intratumoral co-delivery of tumor antigens and TLR agonists, executed via Kim’s nanogel system to ensure synchronized interactions with circulating APCs during lymphatic migration [76]. Lymph node homing employs Laponite-based gel formulations that exploit intrinsic lymphatic tropism, enabling active antigen transport to lymph nodes where resident dendritic cell maturation initiates cross-presentation and CD8+ T cell priming [77]. mmunomodulatory niches utilize silk fibroin scaffolds augmented with STING agonists and ICD inducers, establishing a tripartite signaling environment biomimetic of pathogen-associated molecular patterns (PAMPs) to orchestrate dendritic cell maturation and effector T cell differentiation [78]. These strategies transcend conventional adjuvant stoichiometry by reconstructing the spatial organization inherent to native immune niches.

### 2.4. Overcoming Biocompatibility Barriers via Surface Engineering

Despite significant advancements in hydrogel formulation, clinical translation remains impeded by inherent challenges stemming from uncontrolled polymer degradation and suboptimal tissue interactions. Multiscale engineering strategies are being developed to overcome these limitations by effectively reducing inflammatory responses while simultaneously enhancing the integration of engineered materials with host tissues.

#### 2.4.1. Neutralizing Acidic Degradation Byproducts

PLGA- based hydrogels generate acidic metabolites that lower the local pH, destabilizing antigen conformations and triggering pro-inflammatory cytokine cascades [79]. To counteract this effect, the incorporation of alkaline salts such as Mg(OH)_2_ neutralizes the acidic microenvironment, achieving a 60–70% reduction in cytokine release [80]. Song et al. introduced an innovative system using Poly(ferrocene) aggregates that further enhances this neutralization through oxidative stress-responsive degradation. These aggregates autonomously decompose in H_2_O_2_-rich tumor microenvironments, releasing diferrocene to elevate pH through a positive feedback loop [81]. This self-regulating system not only suppresses tumor growth but also remodels the immunosuppressive microenvironment, demonstrating the therapeutic potential of closed-loop acid-base homeostasis.

#### 2.4.2. Surface Modifications for Tissue Integration

Seamless tissue integration requires the modification of surface properties, including charge, hydrophobicity, and topography, to direct anti-inflammatory macrophage polarization and mitigate foreign body reactions [82]. For instance, Chen et al. revealed that their CA-AuAgNPs-Gel could suppress tumor recurrence without chemotherapy by synergizing antitumor effects with wound healing [83]. PEGylation reduces chitosan’s cytotoxicity by 50–80% while preserving immunostimulatory properties [84]. In addition, high-molecular-weight HA (100–300 kDa) promotes anti-inflammatory CD44 signaling, whereas low-molecular-weight HA activates NF-κB, highlighting molecular weight as a critical design parameter [33]. Collectively, these strategies demonstrate that the precise manipulation of charge, hydrophobicity, and molecular weight enables dual functionality: suppressing foreign body reactions and enhancing immunomodulatory efficacy. Such multiscale control is critical for translating hydrogels from bench to bedside.

#### 2.4.3. Dynamic Mechanics for Physiological Synergy

Achieving physiological synergy requires hydrogels to dynamically match their degradation profiles to the kinetics of tissue repair. MMP-responsive hydrogels developed by Zheng et al. exemplify this principle by incorporating matrix metalloproteinase-cleavable links to adjust degradation rates in concert with wound healing progression [48]. Building on this foundation, oxidative stress-responsive systems introduce spatial-temporal control by remaining stable under normoxic conditions while undergoing rapid dissociation in high ROS environments characteristic of tumor microenvironments [46]. This adaptive mechanism precisely confines drug release to diseased regions, while the material’s evolving architecture sustains its structural integrity amid ongoing tissue remodeling. Crucially, the preservation of structural coherence during tissue remodeling represents a fundamental prerequisite for bridging benchtop discoveries to clinical applications.

## 3. Preclinical Advancements: Mechanistic Insights into Hydrogel-Mediated Immune Reprogramming

This section elucidates the core mechanisms underlying the application of in situ-forming hydrogels in postoperative immunotherapy for breast cancer. We systematically expound on how these hydrogels achieve spatiotemporally controlled drug delivery, thereby orchestrating a cascade from innate immune priming to adaptive immune enhancement, culminating in the metabolic reprogramming of the immune microenvironment. Furthermore, synergistic strategies that integrate hydrogel-based approaches with standard-of-care therapies are explored, and a rigorous preclinical evaluation framework is established, providing a foundation for future translation.

### 3.1. Matching the Postoperative Niche: In Situ Formation and Spatiotemporal Drug Delivery

**Figure 3:**
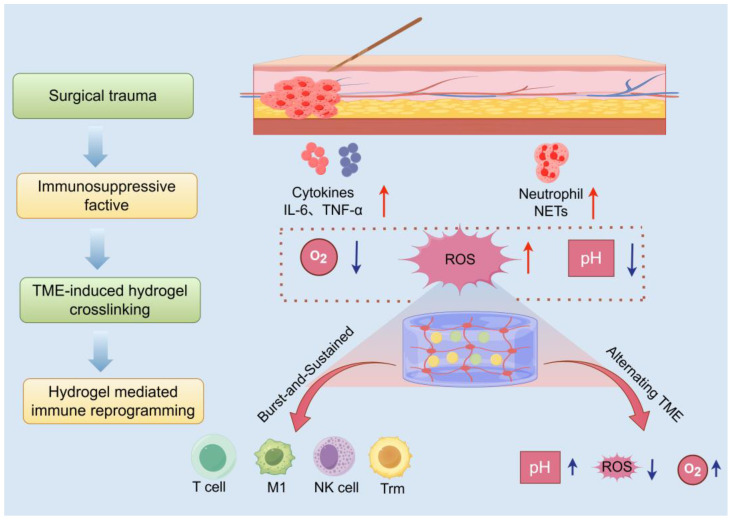
Convergence of hydrogel science and immuno-oncology at the surgical site (generated by Figdraw 2.0).

Surgical resection remains a cornerstone of breast cancer management; however, the unique postoperative environment, known as the ecological niche, poses hidden risks for tumor recurrence and spread. This niche is characterized by acute inflammation stemming directly from surgical trauma, concomitant tissue repair processes, metabolic dysregulation, and pronounced fluctuations in physicochemical parameters. As an intelligent drug delivery platform, in situ-forming hydrogels offer a transformative advantage by precisely matching and proactively responding to this dynamically evolving niche. Figure 3 provides a spatiotemporal mapping of the interactions between the surgical microenvironment, hydrogel deposition sites, and downstream immune activation cascades. This schematic framework highlights the application of the principles of biomaterial design to fine-tune the microenvironment, ultimately potentiating antitumor immunity.

#### 3.1.1. Dynamic Features of the Postoperative Microenvironment

Surgical trauma triggers a robust acute inflammatory response, marked by the extensive release of pro-inflammatory cytokines like interleukin-6 (IL-6) and tumor necrosis factor-α (TNF-α) [85]. Concurrently, hemorrhage, tissue fluid exudation, platelet activation, and the formation of neutrophil extracellular traps (NETs) collectively establish an early immunosuppressive milieu [86]. This setting provides a supportive niche for the survival and immune evasion of residual or circulating tumor cells. Moreover, the surgical site exhibits hypoxia, acidosis, and heightened ROS levels, which are key features exacerbated during wound healing and serve as essential design targets for stimulus-responsive hydrogel systems [85].

#### 3.1.2. Hydrogel Crosslinking Mechanisms and Niche Matching

To counteract the complex challenges posed by the postoperative microenvironment, researchers have engineered advanced in situ-forming hydrogels that are injected as liquid precursors into irregular postsurgical cavities. These formulations rapidly undergo gelation upon exposure to physiological cues such as body temperature or niche-specific signals, with their triggering mechanisms meticulously tailored to exploit distinct features of the postoperative milieu. For example, pH-sensitive bonds, such as acetal/ketal and maleimide groups, utilize the acidic nature of tumor tissues or lysosomal compartments to enable targeted drug delivery [87]. Meanwhile, ROS-labile linkages, including thioketal and borate esters, enable on-demand payload liberation under oxidative stress conditions prevalent at the wound site [88]. Meanwhile, MMP-cleavable sequences integrate enzyme-sensitive peptide motifs directly into the hydrogel backbone, allowing controlled degradation in response to protease activity within the microenvironment [89]. Complementing these chemical strategies, bioadhesive materials such as catechol, chitosan-, or gelatin-based hydrogels actively seal surgical margins, creating a physical barrier that effectively curtails the local dissemination of tumor cells [90].

#### 3.1.3. Matching Spatiotemporal Drug Release Kinetics to Postoperative Immunological Priming

The complexity of hydrogel design lies in its customizable pharmacokinetics, particularly the biphasic release pattern of pulse and sustained release that matches the timing of postoperative immune responses. During the crucial innate immune window (0–72 h post-surgery), hydrogels quickly release immune adjuvants (e.g., TLR agonists) to activate macrophages and dendritic cells, thereby establishing an early antitumor immune advantage [85]. Subsequently, the sustained phase of drug delivery is mediated by mechanisms such as gradual degradation or diffusion-driven release, which ensure prolonged exposure to ICIs, cytokines, or low-dose chemotherapeutics [16]. This extended presentation maintains T-cell activation, reverses exhaustion, and ultimately mitigates late-stage recurrence by reinforcing adaptive immunity.

### 3.2. Reprogramming Innate Immunity: From Immunosuppression to Immune Priming

The innate immune system acts as the body’s primary defense mechanism against pathogens and abnormal cells. However, in the postoperative setting, innate immune cells such as macrophages and DCs often exhibit functional impairment. In situ-forming hydrogel delivery systems mitigate this limitation by delivering immunomodulatory agents with precision to activate and reprogram dormant or co-opted cells, converting an immunosuppressive niche into a highly immune-active environment.

#### 3.2.1. Polarization of Macrophages from M2 to M1

Tumor- associated macrophages (TAMs) generally assume a pro-tumorigenic M2 polarization [91]. It is now understood that hydrogels leverage various methods to convert TAMs into an anti-tumor M1 phenotype, focusing on the targeted delivery of TLR or STING agonists to directly stimulate intracellular pattern recognition [18,85]. Concurrently, chemotherapeutic agents encapsulated within hydrogels induce ICD in tumor cells, releasing DAMPs that further drive M1 polarization. Wang et al. developed an iGEL system, which efficiently releases chemotherapy and TLR7/8 agonists, thereby promoting M1 gene expression and inhibiting M2 markers in bone marrow-derived macrophages [18], validating its potent repolarizing capacity.

#### 3.2.2. Dendritic Cell Maturation and Cross-Presentation

DCs are pivotal in bridging the innate and adaptive immune systems, with hydrogels functioning as danger signal hubs to co-deliver tumor antigens and adjuvants such as R848 and poly I:C [92]. This synergy facilitates DC maturation, as indicated by the upregulation of surface markers (CD80, CD86, MHC-II), and improves cross-presentation efficiency, particularly by activating DC subset 1, which is responsible for cross-presentation. The porous structure of the hydrogel, along with its degradation rate and the size of the nanocarrier particles, collectively influence how antigens are retained, processed, and transported to draining lymph nodes, thereby optimizing the kinetics of T-cell priming [93,94,95].

#### 3.2.3. Neutrophil Regulation and NK Cell Activation

In addition to macrophages and DCs, hydrogels influence other parts of the innate immune system. By embedding DNase I (a NETs scavenger) in the gel, the DNA structure of NETs can be effectively degraded, thereby reducing its negative impact on promoting tumor spread and immune suppression after surgery [96]. Using a hydrogel for the local sustained release of IL-15/IL-15Rα supercomplex or IL-12 can directly increase the cytotoxic function of NK cells against residual tumor cells [97].

### 3.3. Amplifying Adaptive Immunity: From In Situ Vaccination to Durable Memory

Following the successful priming of innate immunity, a critical subsequent challenge is its efficient translation into robust and durable adaptive immune responses, particularly tumor-specific T cell immunity. In this phase, hydrogels serve dual roles as an in situ vaccine factory and a T cell refueling station, aiming to initiate, amplify, and sustain a T cell army capable of eradicating minimal residual disease and preventing recurrence.

#### 3.3.1. In Situ Vaccination and Antigen Supply

The central concept of in situ vaccination leverages the patient’s tumor as an endogenous source of antigens. When combined with postoperative radiotherapy, chemotherapy, or thermal ablation, hydrogels capture substantial amounts of tumor-associated antigens (TAAs) and neoantigens released during tumor cell death [22]. This captured antigen pool serves as the foundational substrate for in situ vaccine generation, directly linking tumor destruction to localized immune priming. To maximize antigen retention, advanced hydrogels employ dual strategies: prolonging entrapment via electrostatic/hydrophobic interactions within the polymer network, and covalent conjugation of high-value antigens using click chemistry to prevent premature diffusion from the immunization site [98]. Crucially, the captured antigens are strategically positioned alongside co-administered immunomodulators, such as STING or TLR agonists, which significantly enhance the effectiveness of antigen presentation [99].

#### 3.3.2. Amplification and Fine-Tuning of T Cell Responses

After being primed, T cells need additional signals for growth and enhanced functionality. Hydrogels provide these signals by continuously releasing key cytokines (IL-2, IL-15) to aid in T cell proliferation and differentiation [100]. This locoregional approach markedly reduces systemic immune-related adverse events (irAEs). In addition, hydrogels enable temporally controlled immunomodulation through the co-delivery of low-dose chemotherapeutic agents, employing a strategy that initially eliminates suppression and then enhances effector function [101].

#### 3.3.3. Induction of Tissue-Resident Memory T Cells (Trm) and Long-Term Protection

The primary aim of cancer immunotherapy is to create lasting immune memory that prevents the recurrence of tumors. Kim et al. designed a thermosensitive LC-Gel for the co-delivery of the oncolytic peptide LTX-315 and CCL21, which triggers the formation of splenic germinal centers and the generation of circulating CD44^+^CD62L^−^ effector memory T cells [102]. Ke et al. improved this strategy by incorporating hemostatic hydrogels with nanoparticle-based drug delivery systems, effectively halting tumor regrowth in treated mice, with survival extending beyond 60 days, compared to 36 days in the control group [103].

**Figure 4:**
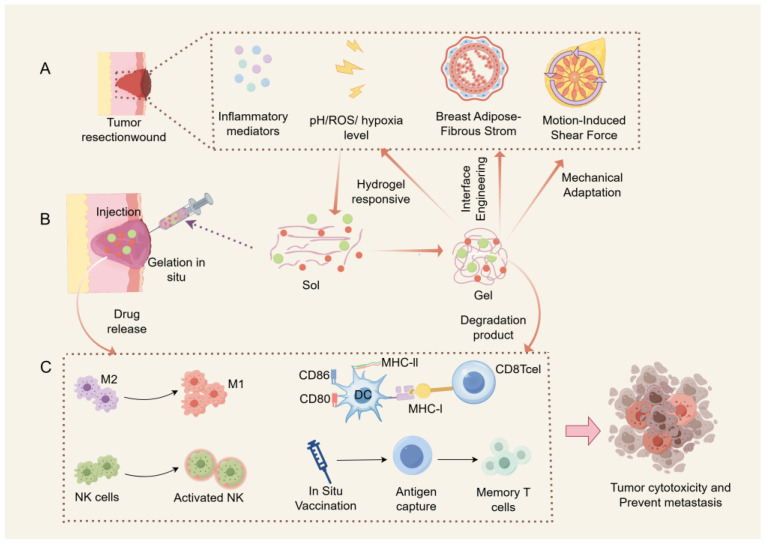
Hydrogel-mediated immune microenvironment engineering in post-lumpectomy cavities: (**A**) pathological microenvironment challenges of post-breast cancer surgery; (**B**) hydrogel Iintervention platform; (**C**) immunopolarization cascade and therapeutic outcome. (Generated by Figdraw 2.0.)

Collectively, these data support the proposed mechanism illustrated in Figure 4, demonstrating how rational hydrogel engineering can transform the immunosuppressive post-surgical niche into an antitumor immunization site.

### 3.4. Material-Mediated Tuning of the Immunometabolic and Physicochemical Microenvironment

Hydrogels serve as versatile platforms that extend beyond drug delivery to actively remodel the immunosuppressive tumor microenvironment (TME) by targeting its metabolic and physical properties

Pathological acidification caused by excessive tumor glycolysis and lactate accumulation severely compromises T cell cytotoxicity [104]. To counteract this hostile microenvironment, hydrogels incorporate buffering agents, such as bicarbonates, or utilize proton-scavenging degradable polymers, like polyesters, to neutralize local acidosis and restore pH homeostasis [105].

In addition, solid tumors exhibit profound hypoxia, a microenvironmental barrier that severely restricts immune cell function. To counteract this challenge, hydrogels implement oxygen-generating strategies by loading catalase or peroxidase-mimetic nanozymes. These enzymes catalytically decompose endogenous hydrogen peroxide in situ, generating nascent oxygen that sustains T cell viability and preserves their effector functions within hypoxic regions [106].

Furthermore, malignant cells exert metabolic pressure on T cells through the competitive consumption of arginine and tryptophan [107]. Hydrogels counteract this by delivering arginase inhibitors or indoleamine 2,3-dioxygenase (IDO) blockers locally, thereby restoring essential nutrient availability and reactivating T cell proliferation and functionality [108]. Supporting this multifaceted approach, a study demonstrated synergistic efficacy by co-encapsulating doxorubicin with kynureninase within hydrogels, achieving both chemotherapy-induced immune activation and reversal of metabolic immune restrictions [101].

### 3.5. Synergistic Strategies: Integrating Hydrogels with Standard-of-Care Therapies

The hydrogel platform is designed not to supplant standard postoperative treatment, such as radiotherapy, chemotherapy, and systemic ICIs, but to function as a potent enhancer, thereby amplifying therapeutic efficacy in breast cancer management.

#### 3.5.1. Synergy with Postoperative Radiotherapy/Chemotherapy

Radiotherapy and specific chemotherapeutic agents like doxorubicin and paclitaxel trigger ICD, leading to the release of DAMPs and tumor antigens. However, these treatments can also exert immunosuppressive effects [109]. In this scenario, hydrogels serve as both a capture reservoir and an amplification hub, isolating ICD-derived antigens and working in synergy with co-delivered immunoadjuvants to convert immunologically ‘cold’ tumors into ‘hot’ ones [101]. Furthermore, delivering chemotherapy locally through hydrogels results in high concentrations within tumors and low systemic exposure, thereby improving ICD effectiveness and reducing damage to peripheral immune organs, such as bone marrow.

#### 3.5.2. Synergy with Systemic ICIs

Despite achieving remarkable successes in certain malignancies, systemic ICIs yield limited response rates in breast cancer and are frequently associated with severe irAEs. Integrating in situ-forming hydrogels with ICIs represents a high-impact strategy to counteract tumor immune evasion. Cheng et al. found that silk fibroin hydrogel vaccines, when used in combination with anti-PD-1 or OX40L in a postsurgical 4T1 TNBC model, effectively stimulated antitumor immunity, inhibited metastasis, and prevented recurrence [78]. In terms of dosing sequences, it is hypothesized that using hydrogels for preactivation followed by systemic ICIs for boosting will create more lasting immune responses than administering them simultaneously. Innovations such as layered hydrogels or microparticle-in-gel hybrids enable sequential release profiles, optimizing timing between immune priming and ICI maintenance [110].

### 3.6. Preclinical Evaluation and Benchmarking: Models, Readouts, and Reproducibility

To advance hydrogel-based immunotherapeutic strategies from bench to bedside, a comprehensive and standardized preclinical evaluation framework is imperative, coupled with rigorous attention to experimental reproducibility. This requirement stems not only from scientific rigor but also serves as the foundation for successful clinical translation.

#### 3.6.1. Animal Model Selection

Model selection is crucial in the study of postoperative breast cancer recurrence. Researchers often utilize syngeneic murine models, including the highly metastatic 4T1 and E0771 models, which effectively mimic triple-negative breast cancer [23,109]. In addition, the MMTV-PyMT transgenic models are frequently employed as they more accurately reflect spontaneous tumorigenesis [111]. Post-resection residual or recurrence models built on these platforms offer clinically relevant assessments of therapeutic efficacy. When investigating drugs targeting human-specific immune epitopes, patient-derived xenograft (PDX) models or humanized immune system-reconstituted mice become necessary, albeit with well-established limitations [112].

#### 3.6.2. Standardized Surgical Protocols

Regardless of model selection, therapeutic efficacy critically depends on aligning localized gel-based interventions with the temporal rhythm of postoperative inflammation. Table 1 presents the harmonized parameters for postoperative studies, highlighting timing as a key determinant of outcome. These principles offer actionable heuristics for clinical dosing schedules, accelerating the translation of preclinical insights into viable therapeutic paradigms.

## 4. Clinical Translation Status and Challenges

The integration of in situ-forming hydrogels into clinical oncology represents a paradigm shift in cancer therapy, merging biomaterial science with precision drug delivery, immune modulation, and multimodal therapy.

Yet, this transformation unfolds unevenly across disease sites. As summarized in Table 2 below, while several hydrogel platforms have reached late-stage clinical development in indications like bladder cancer (UGN-102), esophageal cancer (OncoGel^®^), and melanoma (CHP nanogel), breast cancer lags significantly behind. There are currently no publicly reported ongoing or planned clinical trials, marking a critical gap in the clinical pipeline. This disparity reflects formidable biological hurdles—such as dense post-lumpectomy fibrosis that physically limits hydrogel penetration and creates an immunosuppressive microenvironment [124]—and systemic barriers including a lack of validated biomarkers and consensus endpoints for recurrence prevention [125]. These challenges underscore the complexity of translating promising lab-based technologies into effective bedside interventions.

This chapter analyzes five representative hydrogel platforms: UGN-102 (chemotherapy), OncoGel (chemotherapy), CHP Nanogel Vaccine (immunotherapy), GD2 CAR-T Hydrogel (cell therapy), and Radioactive Hydrogel (radiotherapy), serving as case studies that encapsulate key challenges in translating biomaterial-based therapies from bench to bedside.

### 4.1. Comparative Analysis of Pioneering Platforms

Each platform offers unique insights into the complexities of clinical translation: UGN-102, the first FDA-approved locally delivered chemotherapeutic hydrogel, underscores the value of targeting unmet clinical needs through single-arm trials, albeit amidst methodological scrutiny over trial design rigor [30]. OncoGel’s discontinuation post-Phase II due to lack of superiority over standard care highlights the stringent threshold for demonstrating incremental benefit in settings where conventional therapies dominate. The CHP Nanogel Vaccine, which pioneered in situ immunotherapy, exhibited a limited ability to elicit durable clinical responses despite robust immune activation, illustrating the persistent challenge of converting immunological signals into long-term tumor control [126,127]. The GD2 CAR-T Hydrogel faces regulatory complexities inherent to combination products, emphasizing the need for harmonized frameworks for cell-biomaterial hybrids [128]. Preclinical models of the Radioactive Hydrogel have revealed critical trade-offs between radiation safety and dosimetric precision, necessitating innovative formulation strategies [129].

### 4.2. Strategies for Advancing In Situ-Forming Hydrogels in Breast Cancer Recurrence

**Figure 5:**
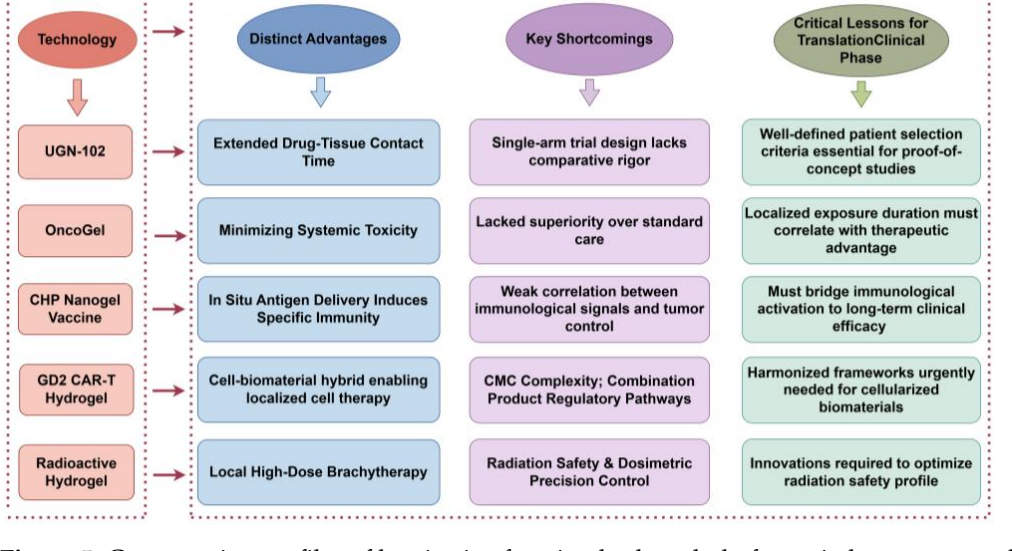
Comparative profiles of key in situ-forming hydrogel platforms in breast cancer therapy. (Generated by Figdraw 2.0.)

Successful clinical translation requires synergistic advancements across three pillars: material innovation, regulatory science, and clinical validation. Figure 5 summarizes the distinct profiles of five representative in situ-forming hydrogel platforms evaluated in breast cancer therapy. This cross-platform comparison highlights their respective therapeutic niches, pitfalls encountered during clinical translation, and actionable insights for overcoming shared barriers.

#### 4.2.1. Limitations of Current Platforms

Current hydrogel platforms, despite their innovative designs, face multifaceted barriers to clinical translation. A primary challenge is manufacturing heterogeneity, manifested as batch-to-batch variability in polymer chain length, crosslink density, and cargo loading efficiency. These issues are exacerbated in biologics-integrated systems (e.g., cell-laden constructs) due to the absence of standardized Good Manufacturing Practice (GMP) protocols tailored for dynamic material systems [130]. Compounding this challenge are immuno-biophysical barriers, including a dense fibrotic stroma and fluctuating interstitial fluid pressure, which severely restrict hydrogel penetration and drug diffusion. Most platforms lack mechanisms to actively remodel these physical obstacles, thereby limiting immune cell infiltration [131]. A critical disconnect persists between preclinical models and clinical outcomes. Animal studies exhibit limited ability in replicating human tumor heterogeneity and wound healing dynamics, and biomarkers predictive of therapeutic response remain unvalidated in breast cancer adjuvant settings [132]. Further complicating translation are regulatory ambiguities stemming from classification conflicts between ‘device’ and ‘biological product’ designations, which obscure regulatory pathways [133], and the lack of consensus on novel endpoints for immunotherapies (e.g., peripheral T-cell clonal expansion) [134]. These interconnected limitations underscore an urgent need for coordinated advancements across material science, manufacturing controls, and regulatory frameworks, goals that will be explored in detail in the following section.

#### 4.2.2. Strategies for Advancing In Situ-Forming Hydrogels

Overcoming these obstacles requires integrated advances in material design, regulatory harmonization, and clinical validation, all of which are essential for translating hydrogel platforms into clinical practice.

Technological refinement must prioritize precise tuning over drug release kinetics to align with tissue pharmacodynamics. Crucially, hydrogel matrices require rational engineering to enhance tissue penetration and disrupt physiological barriers such as dense fibrotic stroma [135]. The CHP Nanogel Vaccine illustrates progress in antigen-targeted lymph node delivery, yet translating this into recurrence prevention demands: (1) improving the conversion of immune activation into measurable clinical endpoints (e.g., reduced recurrence rates) [136]; (2) customizing antigen selection to match tumor heterogeneity, moving beyond shared antigens with suboptimal efficacy [137]; and (3) integrating combination therapies (e.g., PD-1 inhibitors) to counteract immunosuppressive microenvironments [138]. Cross-platform analysis has identified two critical priorities: UGN-102’s trajectory highlights the need for well-defined patient selection criteria for proof-of-concept studies, while OncoGel’s outcomes caution against prolonged localized exposure without demonstrated therapeutic advantage. The manufacturing challenges of GD2 CAR-T Hydrogel underscore the need for standardized protocols for cellularized biomaterials.

Evolving criteria for classifying biohybrid systems demand innovative regulatory approaches, particularly for distinguishing biologics from devices and establishing novel endpoints in vaccine trials [139]. Addressing this requires harmonized frameworks that accommodate the unique characteristics of cell-material combinations, coupled with adaptive clinical trial designs incorporating real-world evidence to accelerate regulatory decision-making.

Linking preclinical immunogenicity to human effectiveness remains challenging, especially in immunotherapies where T-cell activation poorly predicts tumor reduction [136]. Future priorities include developing predictive computational models that correlate in vitro immunogenicity assays with in vivo therapeutic efficacy [140], enabling data-driven optimization of hydrogel formulations. By leveraging cross-disciplinary insights, researchers can develop in situ-forming hydrogels from experimental tools into standard-of-care interventions, transforming perioperative windows into opportunities for curative immunity.

## 5. Future Prospects and Development Directions

Advances in biomaterial design, immunological understanding, and translational engineering increasingly drive the development of in situ-forming hydrogels for postoperative cancer immunotherapy. Research on in situ-forming hydrogels is gradually advancing towards more intelligent, personalized, and therapeutically enhanced approaches.

### 5.1. Artificial Intelligence (AI)-Driven Innovation in the Design of ‘Living’ Hydrogels

Advancing hydrogels from merely reactive smart materials to computational systems that can perform set logic operations and allow for closed-loop control marks the cutting edge of this domain, with AI acting as its revolutionary force. Unlike conventional trial-and-error approaches that demand extensive experimental iterations, AI-driven design frameworks leverage machine learning algorithms to mine vast databases of biomaterial properties and biological interactions [141]. For instance, Liao et al. demonstrated AI-enabled precision engineering by analyzing initial datasets of 180 bioinspired hydrogels through protein sequence pattern recognition, optimizing copolymer chain designs to achieve adhesive strengths exceeding 1 MPa, a performance leap that traditional methods could not match without exhaustive empirical testing [142].

At the molecular level, Jiang et al. combined AI-driven predictions with molecular-scale design principles to identify critical gelation parameters such as crosslinker density and polymer concentration. Their framework accelerated functional optimization of cellulose-based hydrogels for drug delivery applications, highlighting AI’s capability to simultaneously enhance mechanical stability and bioactivity [143]. Similarly, Li et al. underscored AI’s role in multi-parameter optimization, where machine learning models can predict dynamic behaviors like swelling rates and degradation times for novel formulations, reducing experimental failure rates by identifying key variables (e.g., polymer molecular weight, crosslinker type) from high-dimensional data [144].

Critically, beyond material design, AI-driven design frameworks also hold immense potential for achieving dynamic regulation of therapeutic responses. For instance, hierarchical protein hydrogel platforms, engineered with biomimetic tertiary lymphoid structures via AI, can activate pre-existing immune responses within the tumor microenvironment, thereby enhancing immunotherapeutic efficacy [145]. A significant challenge in personalized cancer therapy is a priori prediction of individual patient immune responses. By training AI algorithms on datasets correlating hydrogel properties with immunological outcomes from preclinical models, simulating patient-specific immune reactions to tailor hydrogel formulations is now feasible [146]. In summary, artificial intelligence accelerates material discovery, optimizes treatment strategies, and standardizes evaluation systems, providing critical technical support for the clinical translation of in situ-forming hydrogel-based cancer therapies [147,148]. Future efforts must address challenges such as data standardization and cross-platform integration to fully realize its transformative potential.

### 5.2. Multi-Compartment Architectures for Programmed Immune Synergy

Successful antitumor immunity is a multistep, temporally orchestrated cascade encompassing suppression relief, priming for activation, and memory consolidation. To better simulate and optimize this process, the design of hydrogels is transitioning from a uniform structure to more complex, highly organized multi-compartment configurations. This evolution is particularly crucial for addressing the extreme heterogeneity found in post-surgical cavities, where wound healing rates, vasculature, and residual tumor burden vary significantly across different regions of the same cavity. Leveraging cutting-edge manufacturing techniques like microfluidics, 3D/4D printing, and emulsion templates, researchers can create hydrogels with intricate geometric designs, including core–shell, multi-layered, or side-by-side structures [145,149].

These frameworks enable the spatial segregation of disparate therapeutic agents, thereby preventing unwanted premature reactions between components, such as chemotherapy and immunostimulants. More importantly, they enable precise spatial mapping of the release profile onto the three-dimensional anatomy of the surgical bed. For example, a modular hydrogel vaccine system employs an outer layer for rapid release of chemokines (e.g., CCL21a) to recruit DCs, a middle layer for sustained release of tumor-derived exosomal (Exo) antigens, and an inner layer for continuous delivery of adjuvants such as GM-CSF [150]. This multi-compartment hydrogel integrates functionalities including targeted delivery, immune activation, and tumor microenvironment remodeling through its modular design. It demonstrates unique advantages in addressing critical translational barriers of cancer immunotherapy, such as defective antigen presentation, immunosuppressive TME, and systemic toxicity [150,151]. Its programmable release kinetics and responsive degradation properties further establish a transformative framework for personalized combinatorial therapies.

### 5.3. Accelerating Clinical Translation Through Biomaterial Standardization

Translating in situ-forming hydrogels into clinical applications necessitates a coordinated approach that encompasses manufacturing, regulatory considerations, and the development of biomarkers. Implementing GMP-compliant protocols for the fabrication of hydrogels is essential to guarantee consistent and reproducible results. In addition, engaging with regulatory bodies early in the process, such as through the FDA’s Breakthrough Devices Program, helps to establish a mutual understanding of clinically significant endpoints, including rates of pathological complete response (pCR) [152]. Advanced biomarkers, such as the ratios of intratumoral CD8+ T cells to regulatory T cells (Tregs) and the kinetics of gel degradation that can be tracked using MRI, offer valuable insights into the mechanisms of disease and hold predictive potential for treatment outcomes [153]. Moreover, adaptive trial designs, including basket trials that assess modular gel systems across various solid tumors, facilitate a more efficient evaluation of both safety and efficacy in a wide range of patient populations [154]. This integrated framework bridges biomaterial innovation with clinical validation, paving the way for personalized postoperative immunotherapies.

## 6. Conclusion: Toward a New Vanguard in Postoperative Cancer Care

**Figure 6:**
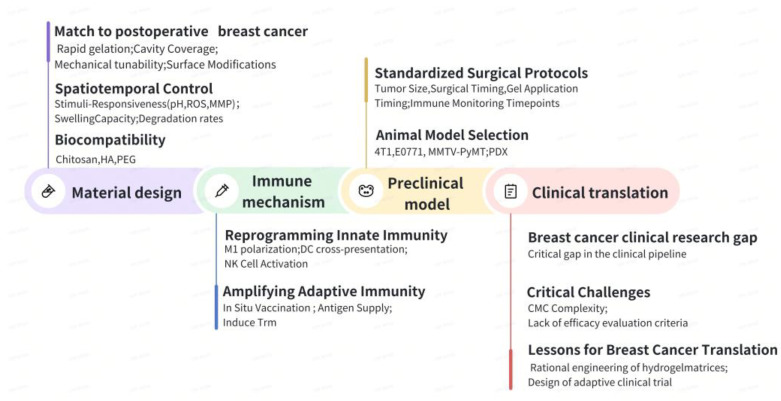
Roadmap for translating material design to clinical impact in postoperative cancer therapy.

The emergence of in situ-forming hydrogels in postoperative cancer therapy stems from their role as active technological linchpins that integrate material design, immunological insights, and clinical needs into a synergistic therapeutic ecosystem. Figure 6 outlines the critical pathway for translating material innovation into clinical impact, mapping the trajectory from immune mechanism and preclinical validation to translational hurdles.

### 6.1. Material Innovation as the Cornerstone

Innovations in hydrogel materials act as the physical support for the treatment framework of cancer after surgery. Their unique ability to gel in place enables minimally invasive delivery into irregular surgical cavities, ensuring they conform to the surrounding tissue and fill any voids, thereby addressing the limitations associated with preformed implants. Importantly, these hydrogels create a localized, high-concentration pharmacochamber within the tumor bed, which helps to prolong the drug’s presence in the area while reducing systemic exposure to the medication. In addition, the integration of TME-responsive elements, such as those sensitive to pH, enzymes, or temperature, enhances their functionality, transforming them into smart, programmable platforms that enable on-demand and sequenced drug release, allowing for precise therapeutic control.

### 6.2. Immune Regulation as the Core

In recent years, hydrogels have undergone a revolutionary transformation, evolving from mere passive carriers to active immune activators. This significant change is supported by two main principles. First, there is temporospatial precision, which involves the engineered sequential release of immunomodulators that effectively convert the immunosuppressive ‘cold’ TME into an activated ‘hot’ state. Second, metabolic reprogramming plays a crucial role; by co-delivering chemotherapeutics alongside metabolic inhibitors, these hydrogels can disrupt the immunosuppressive mechanisms driven by tumors. This innovative approach paves the way for a combinatorial therapy that integrates chemotherapy, immunotherapy, and metabolic intervention, enhancing the overall effectiveness of cancer treatment.

### 6.3. Clinical Benefit as the Goal

All innovations at both the material and immune levels must align with clear clinical objectives. Local immunotherapy utilizing hydrogels demonstrates significant potential for clinical application. Firstly, it effectively inhibits local recurrence and distant metastasis of tumors following surgery, which are primary contributors to treatment failure. Secondly, by targeting highly effective drugs to specific local areas, it significantly reduces the severe toxicities and side effects associated with traditional systemic treatments, thereby enhancing patients’ quality of life. Even more promising is the ability of successful in situ immune activation to induce long-lasting systemic anti-tumor immune memory, known as the Abscopal Effect, which has the potential to eradicate small residual lesions throughout the body.

### 6.4. Future Perspectives: Interdisciplinary Collaboration Drives Translation

Indeed, successful clinical implementation of hydrogel-based therapies requires resolution of critical challenges. Key considerations include preserving structural and functional stability under physiological stressors (such as mechanical forces and enzymatic activity) and minimizing adverse biological responses (including fibrotic encapsulation and chronic inflammation). Achieving full therapeutic potential requires close collaboration among researchers. Materials scientists should prioritize enhancing the biostability of hydrogels in vivo, immunologists must optimize antigen presentation and immune modulation within the matrix, and clinicians must develop adaptive monitoring frameworks for dynamic treatment adjustments. Such multidisciplinary efforts are essential to translate preclinical findings into sustained clinical benefits, thereby realizing the therapeutic potential of precision postoperative immunotherapy.

### 6.5. Epilogue: Toward Precision Postoperative Immunotherapy

In situ-forming hydrogels represent a groundbreaking platform by merging material science, immunology, and clinical strategy. By redefining the end of surgery as the start of therapy, this paradigm shift provides a powerful new approach to tackle breast cancer recurrence and metastasis and suggests a hopeful future for localized immunotherapy in other solid tumors. As biomaterials converge with immunoengineering, these intelligent systems will evolve from experimental curiosity to clinical mainstay, ultimately redefining personalized cancer care.

## Figures and Tables

**Table 1:** Standardized Parameters for Postoperative Cancer Studies in Animal Models.

Parameter	Recommended Range	Rationale	Measurement Methods	Reference
Tumor Size at Resection	≈100 mm^3^	Balance between established tumor and surgical feasibility	Caliper measurements or imaging	[23]
Surgical Timing	12–21 days post-implantation (cell line models)	Allows development of TME	tumor size verification	[23,113,114]
Resection Margins	90–95% resection	Mimics clinical scenario of residual disease	Visual and weight estimation	[23,113,115]
Gel Application Timing	Immediately or Within 3 days post-resection	Maximizes impact on early inflammatory response	Standard operating procedure timing	[23,116,117]
Gel Volume	50–200 μL (mice)	Sufficient to cover cavity without excessive pressure	Calibrated syringe delivery	[23,115],
Antigen Dose	Adjusted according to tumor burden and immunogenicity	Balances immune activation without antigen exhaustion	Protein quantification methods	[118,119]
Study Duration	Short-term efficacy: 2–4 weeks; Long-term protection: 8–12 weeks	Captures both early recurrence and memory formation	Predetermined experimental endpoints	[23,117,120]
Immune Monitoring Timepoints	Day 7 for DC maturation and T cell activation; Days 14–21 for antibody titers and CTL proportions; after day 28 for Trm	Captures key phases of immune response	Flow cytometry, immunohistochemistry	[99,121,122,123]

**Table 2:** Clinical Advancement of Select In Situ-Forming Hydrogel Platforms Across Cancer Types.

Platform	Target Cancer	Clinical Stage	Key Limitation/Breast Cancer Relevance	Reference
UGN-102	Bladder cancer	Approved	Feasibility in a fluid-filled cavity	[29]
OncoGel	esophageal cancer and superficial solid tumors	Phase II discontinued	Insufficient efficacy	[30]
CHP Nanogel	Melanoma and esophageal cancer and prostate cancer	Phase I/II	Low immune response conversion	[31]
None	Breast cancer	Preclinical	lumpectomy fibrosis blocks uniform gelation; lack of standardized efficacy endpoints.	[124,125]

## Data Availability

Not applicable.

## Abbreviations

The following abbreviations are used in this manuscript:

MRD	Minimal residual disease
DCs	Dendritic cells
ICD	Immunogenic cell death
DAMPs	Damage-associated molecular patterns
CHP	Cholesterol-based pullulan
MMP	Matrix metalloproteinase
HA	Hyaluronic acid
PLGA	Poly(lactic-co-glycolic acid)
PEG	Polyethylene glycol
APCs	Antigen-presenting cells
PAMPs	Pathogen-associated molecular patterns
IL-6	Interleukin-6
TNF-α	Tumor necrosis factor-α
NETs	Neutrophil extracellular traps
ROS	Reactive oxygen species
ICIs	Immune checkpoint inhibitors
TAMs	Tumor-associated macrophages
TAAs	Tumor-associated antigens
irAEs	Immune-related adverse events
Trm	Resident Memory T Cells
TME	Tumor microenvironment
GMP	Good Manufacturing Practice
CMC	Chemistry, Manufacturing and Controls
IDO	Indoleamine 2,3-dioxygenase
AI	Artificial intelligence
